# Spiritual Healing: A Review
1*Spiritual Healing: Report of a Clerical and Medical Committee of Enquiry into Spiritual, Faith, and Mental Healing.* Pp. 56. London: Macmillan & Co. Limited. 1914. Price 1s. net.


**Published:** 1914-09

**Authors:** 


### SPIRITUAL HEALING: A REVIEW.[1]

THIS is a very interesting book, not because it brings forward any freshly-discovered facts, but because it is typical of the current thought of the age. At an earlier period of church history it was formally held that all nature is directly subject to the Divine will, and more advanced thinkers, the men who worked out a system of Christian theology, were convinced that this Divine will was directed to the carrying out of some ordered scheme, whose details, indeed, were not completely known to us. This was the "theological" position. The "popular" attitude suspected that there might be some corners which had perhaps escaped the Divine intelligence, and in these obscure recesses it was possible that magic might effect something. The doctrine of an Almighty and All-wise Deity has always contained a serious element of difficulty.

In these later days scientific discovery has revealed an outline, in some places filled in with some detail, elsewhere admittedly rather sketchy, of what we may reasonably regard as an ordered scheme, at any rate so far ordered that it excludes aimless caprice and pure accident. So far this fits in closely with the general theological ideas of such men as Origen, Justin Martyr, St. Augustine, and the scholastics. Admittedly their attempts to fill in details connected with natural science were usually incorrect, but the general idea of an ordered and harmonious scheme, the assumption which still remains at the base of what may be termed orthodox theology, is more or less implied in modern science. But official theology (taking the term to mean the logical development of those traditional teachings which the early

[1] *Spiritual Healing: Report of a Clerical and Medical Committee of Enquiry into Spiritual, Faith, and Mental Healing.* Pp. 56. London: Macmillan & Co. Limited. 1914. Price 1s. net.

Fathers drew partly from revelation and partly from Greek philosophy) has never quite represented the ideas of the numerical majority in the Christian community. Thus popular opinion still inclines to the idea that there may be here and there remote corners to which the Divine will and the Divine wisdom possibly do not extend, items which are too insignificant to work into any scheme, and do not matter much any way.

Now this book before us certainly inclines to such an idea. It seems, at any rate, half implied throughout that it is desirable from the Christian point of view to be able to believe in "miraculous, Divine help from outside, non-physical and outside the ordinary laws of nature" (p. 52). This is expressed by one witness in the statement that "he should like to think it existed" (*id.*), and constantly implied in the reluctance to rule out the "non-physical," presumably from the fear that so doing would hurt the feelings of devout persons. Logically this means that cures can be divided into two classes: the larger one where the cause of healing is known and can be referred to the "ordinary laws of nature;" another class, numerically smaller, in which the cause is not identified, and so the hope is allowable that it may perchance be due to "miraculous, Divine help from outside." In other words, religion is allowed a small residuum in which diagnosis of the cure has failed, and by implication Divine help is only recognised where the method of healing eludes identification. To the scientist this must seem a very unsatisfactory position, for scientific progress is always reducing the number of these elusive phenomena, and he will naturally be inclined to say that the unidentified are simply those which are not yet identified. But to the Christian thinker the position is not simply unsatisfactory, it is suicidal. The more primitive pagan religions may regard their deities as sometimes subject to the laws of nature, sometimes able to elude them; but Christian theology takes ultimate causality as essentially one of the attributes of deity. Physical ordinary laws of nature are laws because they, like creation itself, proceed from the Divine will, and the order observable in nature is there because it reflects a harmonious scheme or idea in the Divine mind, a doctrine already stated by Clement of Alexandria, and in the Epistle to the Hebrews. Whether, therefore, healing of the sick can be directly traced to some known law, that is to say, can be associated with some generalisation drawn from observation of nature, or cannot be definitely traced to any law as yet known, the Primary Cause from which all nature and all nature's laws proceed stands behind as the One responsible. "Ordinary laws of nature," the psychological phenomena of suggestion, etc., are theologically in the same category with the "miraculous." Scientifically they may be divided between the fully-explored field and the *terra incognita, i.e.* that *as yet* unexplored, but the distinction is subjective.

It is true that the Report before us very properly states that Divine power usually works through what it calls "natural channels"—for so we read pages 15, 16—but then the members feel it necessary "to express their belief in the efficacy of prayer" (p. 15). True, theology cannot quite reconcile the effect of prayer with the all-embracing sphere of a Divine will: prophets pray for the coming of Christ, which is already ordained by that will, and Christians pray for the coming of "Thy kingdom," which is equally ordained; but it is perfectly clear that all prayer is subject to the petition "Thy will be done."

Elsewhere this Report seems to base the value of prayer, of Holy Unction, of laying-on of hands, etc., on recorded instances of benefits which have followed, and which cannot be reduced to terms as results from known physical causes. Surely the sounder view, and incidentally the traditional one, is to base the evidence for any means of grace upon the historical fact of their Divine ordinance. Mathematical computation of immediate and material benefits does not appear a very satisfactory line of argument, especially when these benefits are reduced simply to the meagre number which has baffled diagnosis.

There are in this Report very many other points which are extremely interesting. The point which strikes the reader most pleasantly is the readiness to consider the views expressed by the different witnesses, views which could not, in some cases, have quite agreed with those held by members of the committee either in theology or in natural science; and with this the implied admission that knowledge in either field is yet incomplete, an admission already very frankly expressed by St. Paul (1 Cor. xiii. 12).

### Progress of the Medical Sciences.

### MEDICINE.

